# The complete chloroplast genome sequence of *Persicaria perfoliata* (L.) H. Gross: a medicinal plant

**DOI:** 10.1080/23802359.2022.2141079

**Published:** 2022-11-11

**Authors:** Hongsheng Yang, Donghong Yang, Xuewen Yang, Lili Li, Qingbo Zhou, Haitao Cheng, Decai Liu

**Affiliations:** a College of Biology and Agriculture, Jiamusi University, Jiamusi, China; b College of Stomatology, Jiamusi University, Jiamusi, China

**Keywords:** *Persicaria perfoliata*, chloroplast genome, phylogenetic analysis, Polygonaceae

## Abstract

*Persicaria perfoliata* (L.) H. Gross is an herbal medicine with a long history of common use in China. In this study, we sequenced and assembled the complete chloroplast genome sequence of *P. perfoliata* and investigated its phylogenetic relationship in the family Polygonaceae. The total genome size is 160,585 bp in length with 37.96% GC content, consisting of a small single-copy (SSC) of 12,876 bp, a large single-copy (LSC) of 85,439 bp, and two inverted repeats (IRs) of 31,135 bp. The cp genome contains 128 genes, including 35 tRNA genes, eight rRNA genes, and 85 protein-coding genes. The phylogenetic tree showed that *P. perfoliata* was closely related to *P. maackiana*, and *Persicaria* exhibited a closer relationship with *Bistorta* in the family Polygonaceae. This work provides a molecular basis for investigating the evolutionary status, phylogenetic relationships, and population genetics of this species.

*Persicaria perfoliata* (L.) H. Gross 1919 is an annual herbaceous plant belonging to the genus *Persicaria* of Polygonaceae (Hough-Goldstein et al. 2008). It is distributed in the Philippines, Indonesia, North Korea, India, Japan, Russia (Siberia), and China, exhibiting strong survival adaptability (Tan et al. 2013). In China, *P. perfoliata* has a long history being used as a traditional medicine. Modern pharmaceutical studies have shown that *P. perfoliata* has various effects such as antioxidation, anti-tumor, antiviral, anti-inflammatory, anti-liver fibrosis, expectorant, antitussive, and antibacterial activities (Liu et al. 2020). Previous studies on *P. perfoliata* were focused on quality control, pharmacological activities, and phytochemistry (Wang et al. 2009; Xing et al. 2011; Tian et al. 2013). However, up to now for such medicinal plant, the chloroplast genome of *P. perfoliata* has not been analyzed. In this study, we report the complete cp genome sequence of *P. perfoliata* and elucidate its phylogenetic position in the family Polygonaceae.

The healthy and fresh leaves of *P. perfoliata* were collected from Xiaoxing’an Mountains, Heilongjiang Province, China (129°39′E, 46°51′N). The specimens of *P. perfoliata* were deposited at the Herbarium of Jiamusi University under the voucher number XAL2021001 (contact person: Hongsheng Yang and Email: yhongsheng@126.com). Total genomic DNA was extracted by the OMEGA E.Z.N.A.^®^ Plant DNA Kit and sequenced on the Illumina NovaSeq 6000 platform with 150 bp paired-end reads. 72,376,958 raw reads were obtained. Trimmomatic (Bolger et al. 2014) was used to filter the low-quality reads. The remaining 72,257,936 clean reads were used to assemble the cp genome by NOVOplasty v4.3.1 (Dierckxsens et al. 2017) with the complete cp genome of *Persicaria chinensis* (L.) H. Gross (accession number: MN627221) as the reference (Yu et al. 2020). The program PGA (Qu et al. 2019) and GeSeq online (Tillich et al. 2017) performed the annotation followed by manual adjustments. The annotated genomic sequence was submitted to GenBank under accession number OL679838.

The complete cp genome size of *P. perfoliata* is 160,585 bp with 37.96% GC content, which contains two inverted repeats (IRs, 31,135 bp each), separated by small single-copy (SSC, 12,876 bp) and large single-copy (LSC, 85,439 bp) region. The corresponding GC values of the IRs, SSC, and LSC are 41.43%, 33.01%, and 36.18%, respectively. There are 128 annotated genes, including 35 transfer RNA (tRNA) genes, eight ribosomal RNA (rRNA) genes, and 85 protein-coding genes (PCGs). Nineteen genes contain one intron, and four genes (ycf3, clpP, and two rps12) contain two introns.

To investigate the phylogenetic status of *P. perfoliata* in the family Polygonaceae, a phylogenetic tree based on the complete cp genomes of *P. perfoliata* and 29 other species of Polygonaceae was reconstructed using *Plumbago auriculata* as an outgroup (Figure 1). The complete cp genomes were aligned by MAFFT v7.307 (Katoh and Standley 2013), and using the maximum-likelihood (ML) method the phylogenetic analysis was performed by MEGA X (Kumar et al. 2018). The phylogenetic tree showed that *P. perfoliata* was closely related to *P. maackiana*, and *Persicaria* exhibited a closer relationship with *Bistorta* in the family Polygonaceae. Polygonoideae was divided into five tribes including Polygoneae, Rumiceae, Calligoneae, Fagopyreae, and Persicarieae based on the phylogenetic tree, which was consistent with the previous taxonomic treatment (Sanchez et al. 2011).

**Figure 1.**
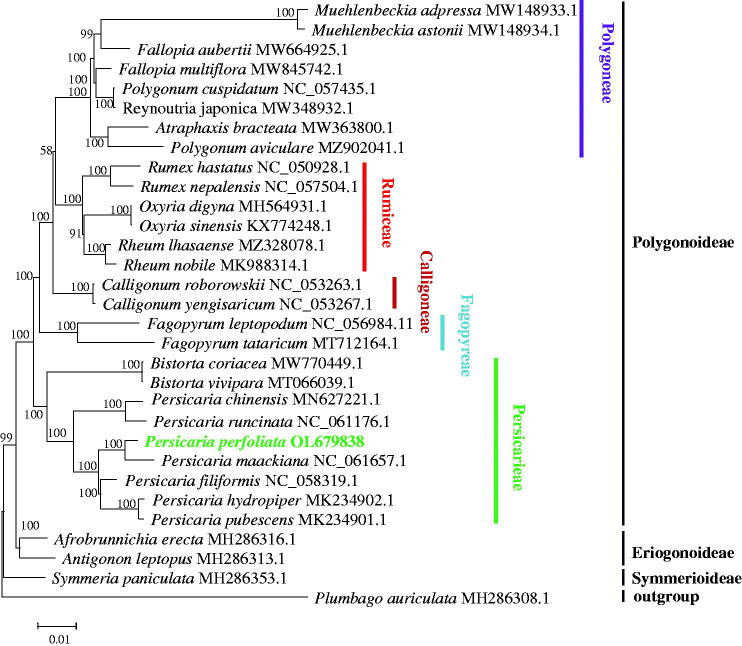
Phylogenetic tree of *Persicaria perfoliata* and 29 other species of Polygonaceae based on the complete chloroplast genome sequence. The species of *Plumbago auriculata* from Plumbaginaceae served as the outgroup. The tree was generated by maximum-likelihood (ML) analysis using MEGA X and the GTR + G+I nucleotide model. The stability of each tree node was tested by bootstrap analysis with 1000 replicates. The numbers on branches were bootstrap support values.

## Ethics statement

The collection of *Persicaria perfoliata* was approved by Tuanjie forest farm, Tangyuan county, Heilongjiang province, China.

## Data Availability

The genome sequence data that support the findings of this study are openly available in GenBank of NCBI at https://www.ncbi.nlm.nih.gov under the accession no. OL679838. The associated BioProject, SRA, and Bio-Sample numbers are PRJNA786268, SRR17152982, and SAMN23638302, respectively.
